# BCMA双特异性T细胞接合器治疗多发性骨髓瘤中国临床专家共识（2025年版）

**DOI:** 10.3760/cma.j.cn121090-20250202-00050

**Published:** 2025-03

**Authors:** 中国医师协会多发性骨髓瘤专业委员会 中华医学会血液学分会浆细胞疾病学组 中国老年医学学会血液学分会

## Abstract

多发性骨髓瘤是一种无法治愈的疾病。针对肿瘤细胞表面抗原的免疫疗法双特异性T细胞接合器显示出良好的临床疗效。然而，双特异性T细胞接合器疗法具有独特的抗骨髓瘤作用机制及不良反应。为进一步规范B细胞成熟抗原双特异性T细胞接合器的临床应用，中国医师协会多发性骨髓瘤专业委员会、中华医学会血液学分会浆细胞疾病学组及中国老年医学学会血液学分会组织专家结合国外最新指南、共识、研究进展及临床实践制订了本专家共识，以期更好地指导临床应用。

## Abstract (en)

Multiple myeloma is an incurable disease. The bispecific T cell engager, one of the immune therapies targeting tumor cell surface antigens, has shown promising clinical efficacy. However, bispecific T cell engager therapy has a unique anti-myeloma mechanism and adverse effects. To further standardize the clinical application of B cell maturation antigen bispecific T cell engager, the Chinese Myeloma Committee of the Chinese Hematology Association, the Plasma Cell Disease Group, Chinese Society of Hematology, Chinese Medical Association and the Society of Hematology of Chinese Geriatrics Association organized experts to formulate the expert consensus by referring to recent domestic and international guidelines, consensus, research progress, and clinical practice, to guide clinical applications better.

多发性骨髓瘤（multiple myeloma, MM）是一种恶性浆细胞疾病，免疫治疗为MM患者带来里程碑式的进步，其中靶向B细胞成熟抗原（B-cell maturation antigen，BCMA）的双特异性T细胞接合器（bispecific T cell engager，BiTE）是复发难治MM（relapse and refractory multiple myeloma, RRMM）重要、极具前景的治疗手段之一[1]。

双特异性抗体指可以同时与两个抗原或抗原表位特异性结合的人工抗体。其中，BiTE是将体内T细胞与表达特定表面标志物的细胞连接起来的双特异性抗体。靶向BCMA和CD3的BiTE，一端结合BCMA，另一端结合T细胞表面的CD3受体，从而将CD3阳性T细胞重定向到表达BCMA的骨髓瘤细胞。这一过程可诱导T细胞的细胞毒性反应，促进骨髓瘤细胞死亡。该机制通过形成肿瘤细胞与T细胞之间的溶解性免疫突触，进一步导致颗粒酶、穿孔素和促炎细胞因子的释放，从而实现HLA非依赖性的T细胞介导的肿瘤细胞杀伤[2]。目前，针对BCMA的BiTE，国内外已有部分药物获得批准，如特立妥单抗（Teclistamab）[3]–[4]和埃纳妥单抗（Elranatamab）[5]–[7]，国产类似药物正在临床试验中。

一、BCMA-BiTE适应证和用法、用量

特立妥单抗在我国获批的适应证为单药治疗既往接受过至少三线治疗（包括一种蛋白酶体抑制剂、一种免疫调节剂和一种抗CD38单克隆抗体）的RRMM成人患者。推荐的起始剂量为0.06 mg/kg（第1天）和0.3 mg/kg（第3天）递增剂量，治疗剂量为1.5 mg/kg（第5天），通过皮下注射给药，后续每周1次，每个治疗周期为28 d。在达到缓解至少6个月的患者中，可考虑将给药频率调整为每2周1次（1.5 mg/kg），直至疾病进展或出现不可接受的不良反应。在MajesTEC-1临床研究中，对于已达到≥完全缓解（CR），且已接受每2周1次给药至少6个月治疗的患者，可考虑将给药频率调整为每4周1次[8]。

埃纳妥单抗是第二款获得FDA批准上市的BCMA/CD3双抗。推荐剂量为第1个周期的第1天和第4天予12 mg和32 mg 2个递增剂量后，患者接受每周1次、每次76 mg皮下注射，28 d为1个周期。6个周期后，持续应答者（≥部分缓解至少持续2个月）改为每2周给药1次[7]。

目前，BCMA-BiTE联合其他药物治疗MM仍处于临床研究阶段，研究方向包括与CD38单抗和免疫调节剂联合治疗等。

二、BCMA-BiTE的关键临床研究结果

MajesTEC-1研究是一项全球多中心、单臂Ⅰ/Ⅱ期临床研究，评估特立妥单抗在RRMM患者中的疗效和安全性。该研究在全球范围内纳入165例患者，随后在中国大陆地区纳入26例患者。全球队列2024年更新的随访结果显示，首次缓解的时间为1.2个月，中位≥CR时间为4.6个月。研究显示，中位随访30.4个月时，总体反应率（ORR）为63％，≥CR率为46.1％，可评估患者的微小残留病（MRD）阴性率为85.7％（48/56）。中位无进展生存（PFS）期和总生存（OS）期分别为11.4个月和22.2个月[8]–[9]。全球队列入组期间与新型冠状病毒感染发病及死亡高峰重叠，排除新型冠状病毒相关死亡影响后的中位PFS期为15.1个月[10]。中国队列26例患者的ORR为76.9％，≥CR率为57.7％。可评估患者的MRD阴性率为90.0％（18/20），12个月时的PFS率为68.0％，OS率为83.5％[11]。在MajesTEC-1全球Ⅱ期扩展队列中，五药难治、高危细胞遗传学和年龄≥75岁患者的ORR和≥CR率与总体人群相当，对于骨髓浆细胞比例≥60％、≥1个髓外浆细胞瘤或国际预后分期Ⅲ期的患者，ORR和≥CR率略低（ORR分别为44.4％、35.7％和35.0％；≥CR率分别为27.8％、17.9％和15.0％），但其24个月的持续缓解率与总体人群相似（分别为42.9％、50.0％和57.1％）[8]。需注意的是，上述亚组患者数量较少，因此仍需要更多证据支持在上述亚组中的治疗效果。

真实世界数据显示特立妥单抗的总体疗效与MajesTEC-1研究相似。一项国际多中心回顾性研究报告了223例RRMM患者的疗效数据，患者既往中位接受治疗线数为6（4～8），其中52％的患者为五药难治，42％的患者曾接受过靶向BCMA治疗，76％（161/212）的患者不符合MajesTEC-1试验的入组条件。研究显示ORR为66％，≥CR率为25％，≥非常好的部分缓解率为59％。中位随访14个月，中位PFS期为8.8（95％ *CI* 5.5～14.0）个月，中位OS期为20个月（95％ *CI* 18个月～未达到），中位缓解持续时间为17个月（95％ *CI* 17个月～未达到）[12]。另一项美国多中心回顾性研究显示中位PFS期为12.0（95％ *CI* 7.1～15.0）个月[13]。

埃纳妥单抗的批准基于MagnetisMM-3研究的结果。该研究纳入的患者均为既往接受过至少一种蛋白酶体抑制剂、免疫调节剂和抗CD38单抗治疗后的难治患者。截至2024年3月更新的随访结果显示，首次缓解时间为1.2（0.9～7.4）个月；中位≥CR时间为6.1（1.2～14.3）个月。中位随访17.6个月，ORR为61％，≥CR率为37.4％，中位缓解持续时间尚未达到，中位PFS期和OS期分别为17.2个月和21.9个月[7],[14]。

三、不良反应

1. 细胞因子释放综合征（cytokine release syndrome，CRS）：CRS是一种全身性炎症反应，在免疫治疗中较为常见，特别是涉及T细胞的治疗。CRS通常发生在免疫细胞被激活后，大量细胞因子（如白细胞介素-6、白细胞介素-10、干扰素-γ等）释放到体内，导致相关临床症状[15]–[16]。目前，已获批准用于MM治疗的BiTE均可能引发CRS（详见表1），发生率为57％～72％，其中大多数为1级或2级反应，3级及以上的发生率较低，为0～2％，且通常只发生在递增剂量阶段或首次全剂量给药时。特立妥单抗引发CRS的中位时间和持续时间分别为2（1～6）d和2（1～9）d[8]；埃纳妥单抗则分别为2（1～9）d和2（1～9）d[7]。

**表1:** BCMA双特异性T细胞接合器主要研究汇总

研究	例数	既往治疗线数	中位随访时间（月）	ORR/≥CR率	中位PFS期（月）	CRS（1～2级/3～4级）	ICANS（1～2级/3～4级）	感染（所有级别/≥3级）	AE停药率
特立妥单抗（MajesTEC-1）[8]–[9]	165	5	30.4	63.0％/46.1％	11.4^a^	72.1％/0.6％	3％/0	79％/55％	4.80％
特立妥单抗（MajesTEC-1中国队列）[11]	26	5	15.0	76.9％/57.7％	12个月PFS率68％	96.2％/0	0/0	96.2％/73.1％^b^	0
埃纳妥单抗（MagnetisMM-1，≥215 µg/kg亚组）[6]	55	5	12.0	63.6％/38.2％	11.8	87.3％/0	–/–	74.5％/27.3％	–
埃纳妥单抗（MagnetisMM-3）[7],[14]	123	5	17.6	61.0％/37.4％	17.2	57.7％/0	4.9％/–	70.7％/48.8％	13.80％

**注** BCMA：B细胞成熟抗原；ORR：总体反应率；CR：完全缓解；PFS：无进展生存；CRS：细胞因子释放综合征；ICANS：免疫效应细胞相关神经毒性综合征；AE：不良反应；–：无数据；^a^MajesTEC-1全球Ⅱ期扩展队列患者入组（2020年3月至2021年3月）与全球新型冠状病毒感染（COVID-19）发病和死亡高峰重叠，去除COVID-19死亡影响后的中位PFS期为15.1个月；^b^造成中国队列感染率高的因素包含COVID-19，2022年12月起中国大陆地区发生了大规模感染，共73.1％的患者感染了新型冠状病毒，其中≥3级COVID-19发生率为53.8％

CRS的临床表现可从轻度输注反应、发热等自限性症状到严重反应，如低血压、呼吸急促、低氧血症等。由于CRS的临床表现缺乏特异性，诊断时需要排除其他潜在的并发症，包括感染、肿瘤溶解综合征、过敏反应等。因此，所有疑似病例应通过详细的病史采集、体格检查及相关实验室检查辅助诊断，如炎症标志物、细菌培养、影像学检查及凝血功能等。对于一线干预后12 h内发热、终末器官不良反应（如缺氧、低血压）等无改善者，需警惕并发噬血细胞淋巴组织细胞增生症/巨噬细胞活化综合征（hemophagocytic lymphohistiocytosis/macrophage activation syndrome，HLH/MAS）。CRS的评估应依据美国移植与细胞治疗协会（American Society for Transplantation and Cellular Therapy，ASTCT）标准[17]。

为了降低CRS的发生率及其严重程度，在BiTE的临床应用中应采取不同的缓解策略，如剂量递增、预防用药和患者监测等。对于大多数BiTE，推荐采用递增剂量方案，以降低CRS发生的风险。在递增给药方案前及首次全剂量给药前，建议使用预防性药物，包括退热剂、抗组胺药、糖皮质激素或托珠单抗。

患者出现CRS症状时，应根据其严重程度采取支持性治疗（如予对乙酰氨基酚、静脉补液及按需吸氧）、托珠单抗和（或）糖皮质激素治疗，同时应考虑暂停或延迟BiTE的给药。CRS的管理可参照ASTCT标准，并根据其严重程度进行分级管理（详见表2）[17]。每次给药后，应评估是否需要应用托珠单抗，最多可在24 h内予3个剂量托珠单抗（每个剂量8 mg/kg），总剂量不超过4个。对于发热和（或）中性粒细减少症患者，建议考虑经验性抗感染治疗。应用糖皮质激素治疗CRS的患者，应考虑预防性抗真菌治疗。

**表2:** 细胞因子释放综合征（CRS）分级管理建议

CRS分级	分级标准	管理建议
1级	发热（体温>38 °C，伴或不伴其他体征），且排除其他发热原因	密切观察； 监护，评估是否存在感染，支持治疗（包括解热镇痛药物），如果持续1级（>24 h且<48 h）和难治性发热则考虑托珠单抗治疗
2级	发热伴低血压（不需应用升压药）和（或）低血氧（需要低流量吸氧）	托珠单抗8 mg/kg； 监护，支持治疗（包括吸氧、补液），如果没有改善，考虑增加糖皮质激素
3级	发热伴低血压（需要应用1种升压药，伴或不伴血管加压素）和（或）低血氧（需要高流量鼻导管、面罩吸氧，但不需要借助机械通气）	托珠单抗8 mg/kg联合地塞米松10 mg，每6 h 1次； 转至监护病房，支持治疗，如果难治，增加地塞米松至每6 h 20 mg静脉滴注；如果病情未改善，可考虑加用阿那白滞素
4级	发热伴低血压（需要多种升压药，但不包括血管加压素）和（或）低血氧（需正压通气，包括持续气道正压通气、气管插管和机械通气）	托珠单抗8 mg/kg联合大剂量糖皮质激素； 转至监护病房，支持治疗，托珠单抗联合大剂量糖皮质激素，可予大剂量甲泼尼龙1 g/d；如果病情未改善，加用阿那白滞素

总体而言，大多数患者在标准支持性治疗下能够快速恢复，但在极少数情况下，重度CRS可能需要密切监测和支持（如血管加压药、阿那白滞素和糖皮质激素），最好在重症监护病房治疗[18]。

2. 免疫效应细胞相关神经毒性综合征（immune effector cell-associated neurotoxicity syndrome，ICANS）：与CAR-T细胞疗法相比，BCMA-BiTE治疗引起ICANS的发生率较低，为3％～5％。当ICANS出现初期症状时，应立即停止使用BCMA-BiTE，并进行神经系统评估，排除其他可能导致神经系统症状的原因。相关检查可包括眼底镜检查（寻找视神经盘水肿）、头部CT或脑MRI（评估脑水肿）、腰椎穿刺（测定颅内压）及脑电图（评估癫痫发作）等。如确诊为脑水肿或颅内压增高，可考虑应用甘露醇治疗。同时，应提供静脉补液、防止误吸、肠外营养等支持性治疗，进行免疫效应细胞相关脑病（immune effector cell-associated encephalopathy，ICE）评分（表3），并根据ICANS的分级进行相应管理（具体分级见表4[19]）。对于接受糖皮质激素治疗的患者，推荐进行预防性抗真菌治疗，且在病情好转时，糖皮质激素应尽快减量。如ICANS对糖皮质激素治疗耐药，可考虑进入临床试验，应用二线方案，如阿那白滞素[20]。

**表3:** 免疫效应细胞相关脑病（ICE）评分

项目	评分标准
定向力	对年、月、城市、医院判定：4分（每项1分）
命名	对3个物体命名（如钟表、笔）：3分（每项1分）
接受指令	遵从指令（如伸出2个手指、闭上眼睛、伸舌）：1分
写作	写出一个标准句子的能力：1分
注意力	反向计数能力，从100、90、80......数到10：1分

**表4:** 免疫效应细胞相关神经毒性综合征（ICANS）管理意见

ICANS分级	分级标准	管理建议
1级	ICE分数：7～9，或意识水平下降：可自行苏醒	高危患者可考虑早期应用地塞米松；考虑非镇静性抗癫痫药物
2级	ICE分数：3～6，或意识水平下降：声音唤醒	地塞米松10 mg，每12 h 1次； 如果48 h后未改善，考虑高剂量地塞米松（每6 h 20 mg）和替代药物（如阿那白滞素）； 开始服用非镇静性抗癫痫药物； 考虑脑电图和CT/MRI成像
3级	ICE分数：0～2，或意识水平下降：仅触觉刺激唤醒，或癫痫发作，表现为如下任一情况：①迅速消退的任何局灶性或全身性临床癫痫发作；②脑电图显示非惊厥性癫痫发作（干预后消退）；③颅内压升高：神经影像学显示局灶性/局部水肿	地塞米松10 mg，每6 h 1次； 如果24 h后未改善，考虑高剂量地塞米松（每6 h 20 mg），高剂量甲泼尼龙（每天1～2 g）和（或）替代品（比如阿那白滞素）； 如果尚未服用非镇静性抗癫痫药物，需要开始服用； 进行CT/MRI成像，考虑脑电图； 考虑采集脑脊液评估其他原因和压力测量； 转移到重症监护室
4级	ICE分数：0，或以下任何一种意识水平下降：①患者无法唤醒或需要强烈或反复的触觉刺激才能唤醒；②木僵或昏迷；或癫痫发作，表现为如下任一情况：a.危及生命的持续性癫痫发作（>5 min）；b.反复的临床或脑电图癫痫发作，两次发作之间未恢复至基线水平；或运动功能改变：深部局灶性动作无力，如轻偏瘫或下肢轻瘫；或颅内压升高/脑水肿，伴有以下体征/症状：神经影像显示弥漫性脑水肿或去大脑或去皮质体位或第Ⅵ颅神经麻痹或视神经乳头水肿或库欣三联征	地塞米松10 mg，每6 h 1次； 对于地塞米松难治性患者，考虑高剂量甲泼尼龙2 mg/kg，每12 h 1次； 对于难治性患者，考虑替代疗法（阿那白滞素）； 如果尚未服用非镇静性抗癫痫药物，需要开始服用； 已经进行CT/MRI成像，考虑脑电图（如果既往没有完成）； 考虑脑脊液检查评估其他原因和压力测量（如果既往没有进行）； 患者应在重症监护室

**注** ICE：免疫效应细胞相关脑病

3. 感染问题：与常规的MM治疗相比，BCMA-BiTE治疗明显增加了感染风险。患者的感染风险与其自身健康状况、疾病本身特点及治疗过程中的多个因素密切相关。已有的临床试验报告显示，接受BCMA-BiTE治疗的患者感染发生率为33％～77％，其中25％～45％的感染为3级或4级感染。值得注意的是，BCMA-BiTE治疗过程中，感染并发症导致的死亡率为6％～12％。根据一项关于全球世界卫生组织药物警戒数据库（纳入188例BiTE治疗后感染病例）研究，开始抗BCMA-BiTE治疗至感染发生的中位时间为44 d。最常见的感染类型为呼吸道感染，包括新型冠状病毒感染（COVID-19）（18％）、肺炎（17％）和脓毒症（12％），其次为巨细胞病毒再激活（9％）、耶氏肺孢子菌肺炎（4％）和其他机会性感染（2％）。此外，约1％的患者发生侵袭性真菌感染[21]。另一项法国多中心回顾性研究报告显示，使用BiTE后，大多数感染发生在呼吸道（58％），其次是尿路感染（15％）、皮肤感染（15％）和胃肠道感染（8％）。在细菌感染中，肠杆菌科占52％；在病毒感染中，呼吸道病毒占63％；而真菌感染中，曲霉菌感染占75％[22]。

感染筛查与监测：在使用BCMA-BiTE治疗前，推荐对RRMM患者进行基线感染状态评估。对于有症状的患者，建议进行病原学检查，包括血液、尿液、痰液和粪便培养等。此外，建议对所有接受BiTE治疗的MM患者进行基线巨细胞病毒、EB病毒、乙型/丙型肝炎病毒和人类免疫缺陷病毒的血清学或分子筛查。对于G试验，常规筛查不推荐，对于IgG型骨髓瘤及部分应用静脉丙种球蛋白的患者，G试验可能存在假阳性，甚至个别患者存在GM试验假阳性。但如果怀疑曲霉病，应进行GM试验检测。如果怀疑真菌感染，必要时可通过影像学检查或纤维支气管镜进行辅助诊断。虽然常规监测肺孢子菌肺炎不被推荐，但既往的临床试验显示，肺孢子菌肺炎的发生率最高可达4.9％，且具有较高的死亡率，因此建议对每例患者进行充分评估，判断是否需要进行肺孢子菌肺炎的影像学及病原学检查。此外，可使用影像学检查评估感染部位和程度，并使用适当的实验室和影像学检查仔细评估是否存在其他感染。在治疗后，可能发生巨细胞病毒、EB病毒和乙型肝炎病毒再激活，因此，应根据患者的具体情况决定是否进行病原体检测[23]。

感染预防：对于接受BCMA-BiTE治疗的患者，推荐进行水痘-带状疱疹病毒和单纯疱疹病毒的预防。常用药物包括阿昔洛韦或伐昔洛韦。在疫苗接种方面，建议无移植史患者每年接种流感疫苗和水痘-带状疱疹疫苗。对于密切接触者，必须定期接种季节性疫苗。推荐对耶氏肺孢子菌进行预防。对于乙型肝炎表面抗原（HBsAg）或乙型肝炎病毒核酸阳性患者，应启动抗乙型肝炎病毒治疗，而单纯乙型肝炎病毒核心抗体阳性患者可选择抗乙型肝炎病毒治疗或进行严密监测。对于预计或已出现粒细胞缺乏≥1周的患者，推荐进行真菌感染的预防。关于是否需要常规使用抗生素进行预防，目前尚有争议。此外，低免疫球蛋白血症的MM患者应考虑应用静脉丙种球蛋白替代补充，以降低严重感染的发生率[23]。

感染治疗：明确病原体后，应进行针对性治疗；对于无法明确病原体的患者，尤其是合并中性粒细胞减少症的患者，推荐使用广谱抗生素，如第三代头孢菌素或碳青霉烯类等。万古霉素应仅在特定情况下（如耐甲氧西林金黄色葡萄球菌感染或导管相关感染）使用。抗病毒治疗方案和持续时间应根据具体病原学类型决定。在抗病毒治疗期间，建议暂时停止BCMA-BiTE治疗，直至感染症状得到临床缓解或病毒载量显著下降。对于侵袭性真菌感染患者，如侵袭性念珠菌病或曲霉病，应根据相应指南进行治疗，治疗期间建议暂停BCMA-BiTE治疗，直至患者症状消退[23]。具体的管理措施参考表5。

**表5:** 接受BCMA双特异性T细胞接合器治疗患者真菌及病毒感染的管理建议

病原体	筛查和监测	预防	治疗
HBV	如果乙肝表面抗原阳性或乙肝核心抗体阳性，则检测乙肝DNA	对DNA检测阳性/病毒血症患者进行抗病毒治疗； 如表面抗原阳性，予抗病毒预防； 如核心抗体阳性，予预防或监测HBV DNA拷贝	恩替卡韦或替诺福韦
HCV	HCV抗体阳性，则检测HCV-RNA	–	根据丙型肝炎基因型选择药物
CMV	怀疑时可行DNA拷贝数检测	–	缬更昔洛韦、更昔洛韦、膦甲酸钠等
EBV	怀疑时可行DNA拷贝数检测	–	利妥昔单抗用于以B细胞受累为主的EBV感染
新型冠状病毒	怀疑时进行核酸检测	遵循当地卫生政策的疫苗接种指导原则进行疫苗接种，建议无移植史的患者接种新型冠状病毒疫苗	奈玛特韦/利托那韦、莫诺拉韦、瑞德西韦等，治疗基于症状和医师评估
流感病毒	仅出现特定症状时进行PCR检测	患者和密切接触者推荐接种流感疫苗	奥司他韦、巴洛沙韦、扎那米韦等
呼吸道合胞病毒或其他呼吸道病毒	仅出现特定症状时进行PCR检测	–	利巴韦林等
单纯疱疹病毒或水痘-带状疱疹病毒	–	对于单纯疱疹病毒或水痘-带状疱疹病毒，推荐预防治疗，且在整个治疗过程中及停药后3个月，推荐的预防治疗包括阿昔洛韦或伐昔洛韦，建议接种疫苗	伐昔洛韦或阿昔洛韦
念珠菌	G试验可能存在假阳性，不常规推荐监测，必要时可通过影像学、纤维支气管镜等检查进行辅助诊断	预期或已经粒细胞缺乏≥1周：氟康唑	氟康唑或棘白菌素类
曲霉菌	如果怀疑曲霉菌病，可进行GM试验检测	伊曲康唑或伏立康唑	伏立康唑、艾沙康唑、伊曲康唑等
耶氏肺孢子菌	不建议常规监测肺孢子菌肺炎感染，但对疑似患者应及时检测	复方磺胺甲恶唑	复方磺胺甲恶唑，若对复方磺胺甲恶唑过敏，可选择阿托伐醌或伯氨喹＋克林霉素

**注** BCMA：B细胞成熟抗原；HBV：乙型肝炎病毒；HCV：丙型肝炎病毒；CMV：巨细胞病毒；EBV：EB病毒；–：无数据

4. 其他不良反应及管理：

（1）低丙种球蛋白血症：RRMM患者常伴随低丙种球蛋白血症，而抗骨髓瘤治疗可能使其加重。低丙种球蛋白血症会显著增加感染风险。对于IgG水平低于4 g/L的患者，推荐应用静脉丙种球蛋白替代治疗，在预防≥3级感染方面有一定效果，对于存在严重或反复感染风险的患者更为重要[24]。需要特别指出的是，IgG型骨髓瘤患者的血清IgG浓度可能高于4 g/L，因此在计算正常IgG水平时，应从总IgG浓度中减去克隆IgG。

（2）血液学不良反应：在BCMA-BiTE治疗的临床试验中出现≥3级血液学不良反应，包括中性粒细胞减少（22％～64％）、贫血（22％～37％）和血小板减少（8.8％～26％）。上述血液学不良反应通常可通过延迟给药和支持性治疗进行管理。对于3级中性粒细胞减少（中性粒细胞绝对计数<0.5×10^9^/L）患者，应暂停BCMA-BiTE治疗，并考虑使用粒细胞集落刺激因子治疗，直至中性粒细胞绝对计数≥0.5×10^9^/L；对于4级中性粒细胞减少或发热性中性粒细胞减少，应暂停治疗并使用粒细胞集落刺激因子，直至中性粒细胞绝对计数≥1×10^9^/L。需要特别注意的是，在CRS发生期间，应避免使用骨髓生长因子，尤其是粒细胞-巨噬细胞集落刺激因子。对于HGB<80 g/L患者，应暂停治疗，并考虑输血或应用促红细胞生成素，直到HGB≥80 g/L；对于PLT低于25×10^9^/L或PLT为（25～50）×10^9^/L且伴出血的患者，应暂停BCMA-BiTE治疗，直至PLT≥25×10^9^/L且无出血证据[19]。

（3）免疫效应细胞相关噬血细胞淋巴组织细胞增生症样综合征（immune effector cell-associated hemophagocytic lymphohistiocytosis-like syndrome, IEC-HS）：IEC-HS是一种诊断困难且危及生命的并发症，已在接受CAR-T细胞疗法的患者中逐渐增多。BiTE与CAR-T细胞疗法具有相似的免疫激活机制，尽管目前尚未见到与BiTE治疗相关的IEC-HS病例，但临床医师应保持高度警觉。如果怀疑患者出现IEC-HS，应考虑进行免疫抑制治疗，包括使用糖皮质激素、依托泊苷或IL-1受体拮抗剂等[25]。

四、总结

针对BCMA的BiTE是RRMM治疗的新利器，但有其独特的安全性问题及使用注意事项，随着BCMA-BiTE在我国的广泛应用，临床医师用药经验将不断丰富，会为临床应用提供更多有价值的参考。
